# Longus Colli Tendinitis: Analysis of MRI and Clinical Features With Predictive Pain Risk Model Development

**DOI:** 10.1155/prm/9211904

**Published:** 2025-09-11

**Authors:** Yanqiang Qiao, Yue Qin, Gang Xiao, Lijun Zhang, Jite Shi, Shaohui Ma, Ming Zhang, Wen Gu

**Affiliations:** ^1^Department of Medical Imaging, The First Affiliated Hospital of Xi'an Jiaotong University, Xi'an, Shaanxi, China; ^2^Department of Radiology, Xi'an Daxing Hospital, Xi'an, Shaanxi, China; ^3^Department of Emergency, Xi'an Daxing Hospital, Xi'an, Shaanxi, China; ^4^College of Medical Technology, Shaanxi University of Chinese Medicine, Xianyang, Shaanxi, China

**Keywords:** longus colli tendinitis, magnetic resonance imaging, model, pain

## Abstract

**Objectives:** Longus colli tendinitis (LCT) is a rare, self-limiting disease primarily characterized by neck pain. This study is to investigate and analyze the imaging and clinical features of LCT and to develop a predictive model for pain risk in LCT based on these features.

**Methods:** This study included 35 patients with LCT enrolled between January 2017 and December 2024. Radiological features, laboratory indicators, and clinical profiles were systematically analyzed. We stratified LCT patients into high-risk (*n* = 20) and low-risk (*n* = 15) groups based on pain intensity and duration. Nomograms were developed using logistic regression models, with feature selection performed via the least absolute shrinkage and selection operator method. Model performance was evaluated through discrimination (Harrell's C-index) and calibration (calibration plots), with internal validation conducted via bootstrapping. A clinical impact curve was used to assess the model's clinical usefulness.

**Results:** MRI features of LCT included average lesion width of 6.13 mm, length of 64.00 mm, circumference of 134.52 mm, and area of 230.64 mm^2^. Clinically, LCT patients exhibited elevated white blood cell counts, neutrophil counts, hsCRP levels, and IL-6 levels. Feature selection revealed that the lesion area could predict pain risk in LCT patients, which was used to construct a predictive model. The model demonstrated a C-index of 0.93 (95% CI 0.84–0.99). Internal validation confirmed the model's robust performance, with a C-index of 0.93 (95% CI 0.83–0.99).

**Conclusion:** LCT possesses distinct imaging and clinical features. Utilizing these features enables effective prediction of pain risk, thereby assisting clinical decision-making.

## 1. Introduction

Longus colli tendinitis (LCT), also known as retropharyngeal calcific tendinitis or prevertebral calcific tendinitis, is a rare and benign condition that typically presents as an acute episode [1, 2]. LCT was first described by Hartley in 1964 [3]. The pathogenesis was confirmed in 1994 by Ring et al. as calcium hydroxyapatite crystal deposition within the superior oblique fibers of the longus colli tendon [4]. This deposition triggers an inflammatory response, leading to symptoms such as severe neck pain, limited neck movement, and odynophagia [4]. LCT, a relatively rare condition, frequently goes unrecognized or is misdiagnosed, often being mistaken for retropharyngeal abscess, meningitis, spondylodiscitis, or neoplastic lesions due to overlapping clinical and imaging features [5, 6]. Such misdiagnoses may lead to unnecessary antibiotic administration, invasive diagnostic procedures, or even surgical interventions.

The clinical evaluation of LCT primarily involves imaging examinations and laboratory blood tests. Laboratory analyses often reveal elevated inflammatory markers and increased white blood cell counts, indicative of an inflammatory process [2]. Imaging examinations involve magnetic resonance imaging (MRI), computed tomography (CT), and digital radiography (DR), with MRI being particularly advantageous in displaying lesions.

In clinical practice, the treatment of LCT, a benign self-limiting condition, includes but is not limited to antibiotics, nonsteroidal anti-inflammatory drugs (NSAIDs), and corticosteroids [7–9]. However, symptoms of LCT generally subside naturally within 2 weeks [7]. Consequently, there may be overtreatment of LCT, which can lead to unnecessary suffering for patients and a waste of resources.

However, despite increasing awareness of LCT, the current research has primarily focused on case reports and descriptive analyses of clinical and imaging features [10–12]. There is a lack of systematic investigation into how quantitative imaging parameters and laboratory inflammatory markers relate to clinical severity, particularly pain intensity. Moreover, to date, no predictive models have been developed to identify patients at high risk for severe pain outcomes. This gap limits early risk stratification and individualized management in clinical settings.

To address these limitations, this study aimed to systematically extract and analyze the imaging and laboratory features of LCT patients and to develop a predictive model for identifying patients at higher risk for severe pain outcomes. By enabling early risk prediction, this model may assist clinicians in optimizing treatment decisions and avoiding unnecessary interventions for low-risk patients.

## 2. Materials and Methods

### 2.1. Data Collection and Study Population

This study received approval from the Medical Ethics Committee of Xi'an Daxing Hospital (application ID: No. Dxll2017-109). Procedures adhered to relevant guidelines and regulations and to the Declaration of Helsinki. Written informed consent was obtained from every participant after the study had been fully explained.

Between January 2017 and December 2024, patients with LCT were consecutively enrolled at Xi'an Daxing Hospital. Demographic characteristics, clinical information, and laboratory measurements were collected for all participants.

The inclusion criteria were as follows: (a) age between 18 and 70 years and (b) a confirmed clinical diagnosis of LCT [13]. Exclusion criteria encompassed (a) coexisting cervical conditions (such as infection, cervical spondylosis, or neoplasms), (b) current use of immunosuppressive therapy, (c) ongoing infections or malignancies;, (d) refusal to participate, (e) pregnancy, and (f) contraindications to MRI (e.g., metallic implants and claustrophobia).

After applying the inclusion/exclusion criteria and eliminating incomplete questionnaires or MRI data with inadequate quality, a total of 35 patients with LCT were included in the final analysis.

### 2.2. Demographic Data Collection, Survey, and Assessments

Demographic and clinical characteristics—including age, sex, height, weight, and symptom duration—were documented for each participant. Laboratory assessments comprised measurements of white blood cell count, neutrophil and lymphocyte levels, eosinophils, basophils, high-sensitivity C-reactive protein (hsCRP), and interleukin-6 (IL-6). All blood samples were processed using standardized protocols in the same clinical laboratory to ensure consistency.

To assess pain severity, participants rated their discomfort using a visual analog scale (VAS), ranging from 0 mm (indicating no pain) to 100 mm (representing the most severe pain imaginable). During the assessment period of 1 week, patients with an average VAS pain score greater than 30 mm and persisted for more than 1 week were classified as high-risk pain. This criterion reflects that their pain intensity reached at least a moderate level [14]. The assessment period for high-risk pain was set at 1 week, as previous reviews have indicated that while the majority of patients with LCT experience symptom relief within 1 week, a minority of severe cases exhibit symptoms that persist for more than 1 week [15].

### 2.3. Image Acquisition

Initial imaging assessments were conducted using either standard lateral cervical radiographs (model 1000D, WanDong Medical, Beijing, China) or CT scans (Ingenuity Core 128, Philips Medical Systems, Amsterdam, Netherlands). Patients were scanned in the supine position, covering the region from the atlanto-occipital junction to the upper thoracic spine. Imaging settings included a tube voltage of 120 kV, tube current of 250 mA, slice thickness of 1 mm, and a pitch of 0.8.

Subsequently, all participants underwent MRI using a 1.5T clinical system (MAGNETOM Area, Siemens AG Healthcare Sector, Erlangen, Germany) equipped with a 16-channel head coil. MRI acquisitions consisted of a T2-weighted Dixon sequence, T2-weighted imaging (T2WI), and T1-weighted imaging (T1WI). Detailed parameters for each MRI sequence are summarized in Table S1.

**Figure 1:**
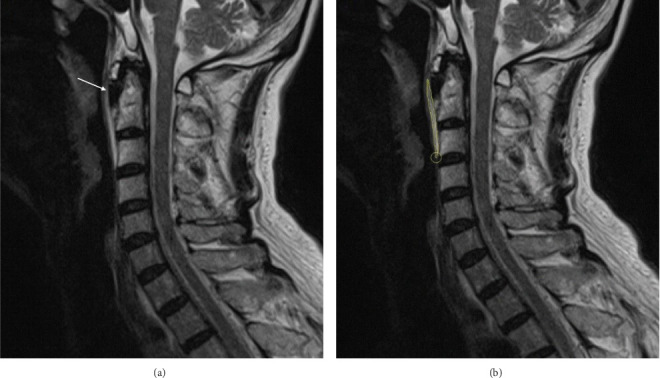
(a) Cervical MRI shows calcification of the longus colli muscle at the level of C1 and C2 (arrow heads). (b) ROIs are delineated to outline the extent of the lesion (edema in the prevertebral soft tissues). ROIs, regions of interest.

Two experienced radiologists independently reviewed the MRI images to exclude cervical lesions and other organic pathologies. Regions of interest (ROIs) were manually delineated on the T2-weighted images to identify LCT lesions (Figures 1(a) and 1(b)). The radiologists were blinded to the patients' clinical pain scores and group assignments during ROI delineation to minimize observer bias. T2WI was selected for ROI delineation due to its superior soft tissue contrast and clearer visualization of inflammatory edema and lesion boundaries in LCT. The delineation results were subjected to interrater reliability analysis using intraclass correlation coefficients (ICC). The lesion area, length, width, and circumference were subsequently calculated based on the delineated ROIs.

### 2.4. Statistical Analysis

Data analyses were implemented with R software (Version 4.2.3; https://www.R-project.org). The primary indicator for sample size was determined as lesion width based on prior study [2]. Sample size was estimated in G∗Power 3.1 (https://www.gpower.hhu.de/), targeting 90% power at *α* = 0.05 and, based on the anticipated effect size, increased by approximately 10% to allow for attrition. Consequently, at least 13 participants were needed per group.

Group comparisons between high-risk and low-risk LCT pain cohorts used the two-sample *t* test for normally distributed continuous data and the Mann–Whitney *U* test for non-normal data. Categorical variables were reported as *n* (%) and evaluated using the *χ*^2^ test, while continuous data were summarized as mean and standard deviation (SD) (normal) or median and interquartile range (IQR) (skewed). The Kolmogorov–Smirnov test was used to assess data normality, while Levene's test evaluated the homogeneity of variances. Multicollinearity among variables was examined using the variance inflation factor (VIF). A *p* value < 0.05 was considered statistically significant.

Least absolute shrinkage and selection operator (LASSO) regression was applied to select key predictive features across demographic, clinical, and imaging domains. Variables with nonzero coefficients after penalization were retained for further modeling. We then conducted univariate logistic regression to examine associations between individual predictors and the likelihood of severe outcomes. After LASSO-based feature selection, a multivariable logistic regression model was fitted. Nomograms were developed to visualize the predicted probability of high-risk pain outcomes in LCT patients. In this study, a similar approach to that used in our previous nomogram development for chronic kidney disease-associated pruritus was adopted for variable selection and model development [16].

Model performance was evaluated by the concordance index (C-index) and the area under the receiver operating characteristic curve (AUROC). Internal validation was conducted with 1000 bootstrap resamples to assess model stability. Calibration curves and confusion matrices were generated to evaluate the model's agreement and predictive classification performance. Additionally, a clinical impact curve was plotted to explore the real-world utility of the LCT risk prediction model in clinical decision-making.

## 3. Results

### 3.1. Demographic, Clinical, and Imaging Data

In total, 35 participants were enrolled, with the high-risk population comprising 57.14% (*n* = 20) and the low-risk population comprising 42.86% (*n* = 15), and complete data were available for all participants.  Table 1 summarizes the participants' demographic, imaging, and clinical features.

The average age of the LCT patients was 49.14 years (SD 16.06), with no significant difference between the high-risk and low-risk groups. The cohort comprised 62.86% males and 37.14% females. A higher proportion of males was observed in the high-risk group (70.00%) than in the low-risk group (53.33%), although this difference was not statistically significant. Participant height and weight also showed no significant differences between groups.

**Figure 2:**
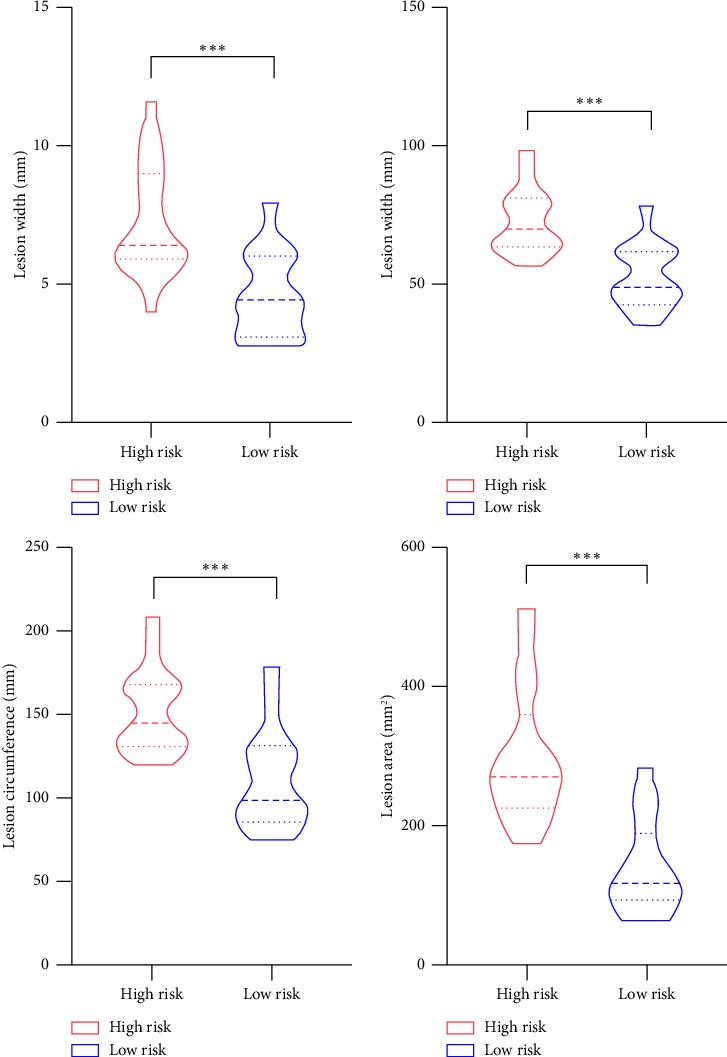
Comparison of imaging features of LCT between the high-risk group and the low-risk group. LCT, longus colli tendinitis. After Bonferroni correction, *α* = 0.0125 (0.05/4). ^∗∗∗^*p* < 0.001.

Patients with LCT demonstrated varying degrees of imaging feature abnormalities. According to Figure 2, lesion width, length, circumference, and area were all significantly greater in the high-risk group than in the low-risk group (*p* < 0.001 for each). Inter-rater reliability for lesion measurements was excellent. The ICC values for width, length, circumference, and area were 0.955 (95% CI: 0.913–0.977), 0.956 (95% CI: 0.914–0.977), 0.896 (95% CI: 0.804–0.946), and 0.901 (95% CI: 0.801–0.951), respectively.

Overall, patients with LCT exhibited average white blood cell counts (reference range, 3.5–9.5 10^9^/L), neutrophil counts (reference range, 1.8–6.3 10^9^/L), hsCRP levels (reference range, < 6 mg/L), and IL-6 levels (reference range, < 7 pg/mL) that were above the reference range. Furthermore, white blood cell count, neutrophils count, and levels of in hsCRP and IL-6 were significantly higher in the high-risk group (*p*=0.007, *p*=0.005, *p*=0.002, and *p*=0.003, respectively). Conversely, lymphocytes, eosinophils, and basophils did not show significant differences between the groups.

### 3.2. Feature Selection

**Figure 3:**
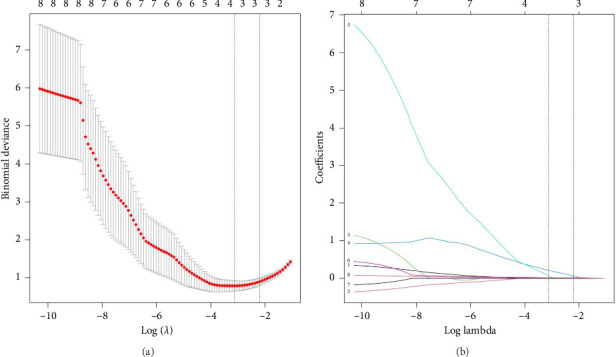
Feature selection using the LASSO model. (a) 10-fold cross-validation identified the penalty; three predictors were retained under the one-standard-error rule at the minimum. (b) LASSO coefficient profiles for the 8 candidate features. LASSO, least absolute shrinkage and selection operator.

Features with significant between-group differences were entered into LASSO for analysis (see Table 2). Following penalization, the initial set of 8 candidate variables was reduced to 3 predictors with nonzero coefficients (Figures 3(a) and 3(b)). The selected features included lesion length, lesion area, and neutrophil count. These variables were subsequently incorporated into the logistic regression model for risk prediction.

Table 2 presents the results of the univariate logistic regression analysis assessing various risk factors associated with high-risk outcomes in patients with LCT. Lesion width, lesion length, lesion circumference, and lesion area were all significantly associated with higher risk outcomes, indicating that larger lesions are linked to increased risk. In terms of clinical features, white blood cell counts and neutrophil count also showed significant associations, reflecting that elevated inflammatory markers predict higher risk in LCT patients. Although hsCRP levels approached significance, IL-6 levels did not demonstrate a significant relationship.

### 3.3. Development of Model

Based on the results of LASSO feature selection, three candidate predictors (lesion length, lesion area, and neutrophil count) were initially identified. After multivariate logistic regression adjustment, lesion area and neutrophil count remained in the final model, while lesion length was excluded due to its nonsignificant contribution after accounting for correlated variables. The regression analysis revealed that the lesion area was significantly associated with an increased risk of severe outcomes, with an odds ratio (OR) of 1.04 (95% CI: 1.01–1.07; *p*=0.014). Neutrophil count showed a trend toward association but did not reach statistical significance (OR: 1.62, 95% CI: 0.99–3.31; *p*=0.100). The detailed results are presented in Table 3.

**Figure 4:**
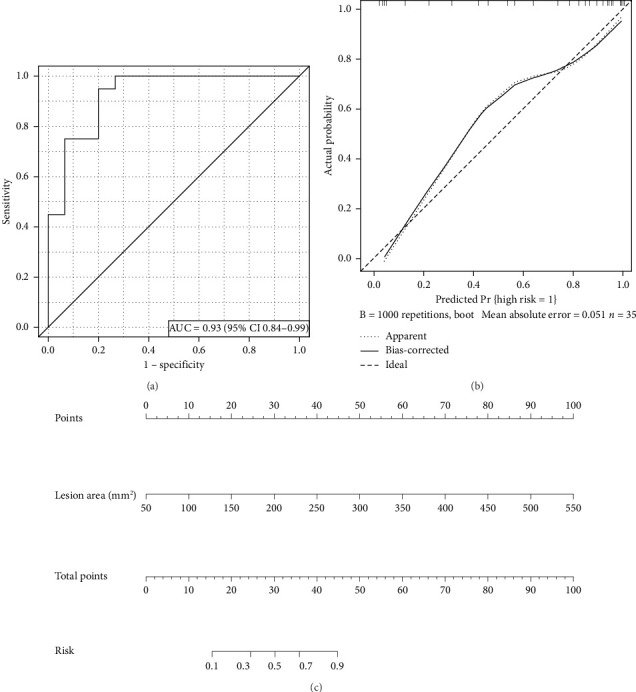
Model performance of the LCT risk model. (a) ROC curve of the model in which the AUROC was 0.93 (95%CI 0.84–0.99). (b) The calibration curves of the model for LCT risk. (c) Nomogram for predicting pain risk of LCT. The diagonal dotted line indicates perfect calibration, and predicted probabilities closely match observed values. In the nomogram, points are read from the top row for each characteristic; summed points give a total score corresponding to the probability of pain risk. AUROC, area under the receiver operating characteristic curve; LCT, longus colli tendinitis; ROC, receiver operating characteristic.

The nomogram was used to evaluate the pain risk of LCT based on the lesion area (shown in Figure 4(c)). The nomogram lists point assignments for individual characteristics on the top row. Summing these produces the total points, corresponding to the probability of pain risk.

### 3.4. Model Assessment and Validation

**Figure 5:**
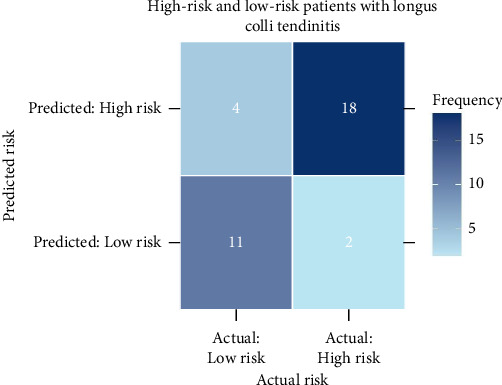
Confusion matrixes of the classification models for LCT risk prediction. This matrix displays the classification outcomes of the LCT risk prediction model. The matrix illustrates the model's performance across various actual and predicted risk levels. LCT, longus colli tendinitis.

**Figure 6:**
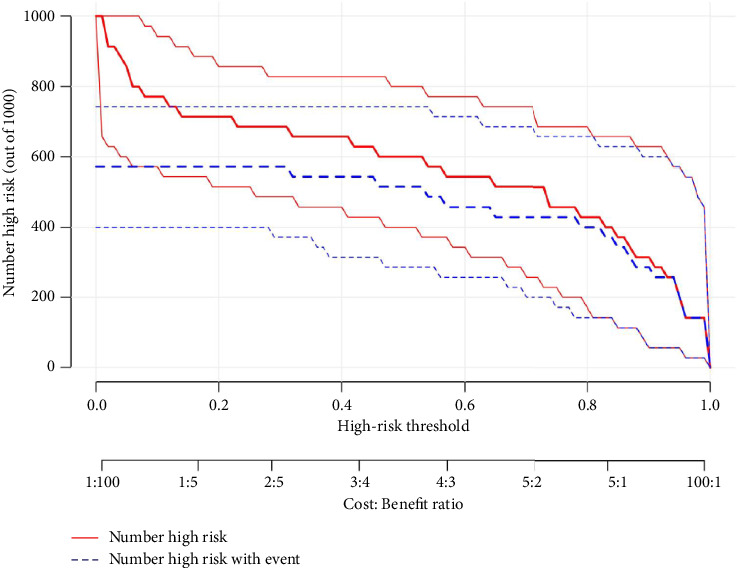
Clinical impact curve for LCT. For a hypothetical cohort of 1000 individuals, the red line indicates the number classified as high risk across threshold probabilities, whereas the blue line shows the actual number of high-risk patients. LCT, longus colli tendinitis.

Model performance was assessed for discrimination and clinical usefulness, with internal validation by bootstrap resampling (1000 iterations). Discrimination was high: the AUROC for the LCT high-risk model was 0.93 (95% CI, 0.84–0.99) (as shown in Figure 4(a)), and C-index was 0.93 (95% CI, 0.84–0.99). Bootstrap validation yielded a similar C-index of 0.93 (95% CI, 0.83–0.99). Calibration indicated good agreement between predicted and observed probabilities (shown in Figure 4(b)). Additionally, the confusion matrix visualizes the performance of the risk prediction model for LCT (shown in Figure 5). Finally, Figure 6 illustrates the clinical impact curve for LCT risk prediction, comparing the predicted high-risk cases (red curve) to actual high-risk events (blue dashed curve) across various thresholds in a cohort of 1000 patients.

## 4. Discussion

In this study, we analyzed the imaging and clinical features of 35 LCT patients and developed a model to predict the risk associated with LCT based on these observations. We identified varying degrees of abnormalities in both imaging and clinical features, which enhance our understanding and diagnosis of LCT. Furthermore, we discovered that the area of the lesion evident in imaging studies can be utilized to predict the severity of future disease progression. This model can assist clinicians in identifying and preemptively treating patients at higher risk.

LCT is considered a rare disease, and a review of the literature reporting cases of RCT from 1990 to 2020 revealed a total of 116 cases reviewed [17]. Gilad et al. have reported an annual incidence of LCT as 0.5 cases per 100,000 person-years, suggesting that LCT may not be as rare as previously thought [7]. The reason for this could be due to the self-limiting nature of the disease, with some patients possibly not seeking medical attention [1]. Additionally, the scarcity of research and lack of awareness about the disease contribute to a high misdiagnosis rate [18]. However, in recent years, with increasing public awareness of personal health and the widespread availability of imaging studies, the detection rate of LCT may have increased.

The incidence of LCT exhibits no gender predilection [1], and the majority of patients in previous studies were aged between 30 and 60 years [19, 20]. In the current study, the mean age of participants was 49.14 years with a SD of 16.06. It has been postulated that the classic clinical triad of this condition includes neck pain, neck stiffness, and odynophagia, although discrepancies exist in the clinical presentations reported in the literature [5, 17, 21]. In our study, nearly all patients reported neck pain, more than half experienced neck stiffness, and less than half had odynophagia.

Imaging diagnosis of LCT is primarily conducted through DR, CT, and MRI [22]. On DR, the typical imaging finding for LCT is swelling of the prevertebral space. On CT scans, prevertebral fluid accumulation and swelling of the longus colli muscle can be identified, with calcifications in the part of the longus colli muscle considered to be pathognomonic of LCT [4, 23]. MRI is more sensitive in detecting abnormalities in soft tissues. On T2WI MRI, edema within the prevertebral muscles presents with a hyperintense signal [9, 17, 24]. Overall, DR and CT scans are readily accessible and provide a good initial diagnosis for LCT [24, 25]. MRI offers high spatial resolution and can provide a wealth of imaging features of LCT, which is beneficial for assessing the disease in later stages.

Therefore, in this study, we measured lesion dimensions such as length, width, circumference, and area on MRI images of LCT patients. After feature selection, we found that the lesion area has a good predictive value for the severity of the disease. This result is likely because the lesion area can reflect the length, width, and circumference of the lesion to a certain extent. Similarly, a 2018 study on LCT measured the retropharyngeal space at the C3 level using cervical simple radiography and found an increase in the retropharyngeal space in LCT patients [2]. Compared to this previous research, our study utilizes MRI imaging to measure lesion features, providing more accurate and comprehensive information than measurements taken with cervical simple radiography.

Laboratory findings reported for patients with LCT primarily focused on elevated white blood cell counts and increased level of C-reactive protein [1, 15, 26]. Additionally, our study noted varying degrees of increases in neutrophils and IL-6 levels. Abnormalities in neutrophils have been reported in previous studies [27]; however, changes in IL-6 levels in LCT are rarely documented. As a key proinflammatory cytokine, IL-6 plays an important role in acute phase responses, soft tissue inflammation, and musculoskeletal pain, which may partly explain its elevation in some LCT patients [28]. Overall, LCT presents with relatively mild inflammatory responses, with only modest increases in inflammatory markers. Some of these markers may provide indications of disease severity.

Given the findings of LCT imaging and clinical features, we used these features to predict which patients are at pain high risk (experiencing from prolonged moderate to severe pain). The results indicated that the lesion area has a predictive value for the intensity of patient pain. There are significant differences in pain intensity and duration of symptoms among patients with LCT [19, 29]. Therefore, in actual clinical practice, some LCT patients exhibit mild symptoms that resolve shortly, yet treatments involving corticosteroids and antibiotics may lead to overtreatment, waste of medical resources, and unnecessary distress for the patients [30]. Therefore, the predictive model can be used for early identification of high-risk patients.

First, although the sample size in this study is the largest to the data among similar studies, we share the same perspective as previous research that the incidence of LCT remains underestimated. Therefore, the collection of more samples would be beneficial in further understanding this condition. Additionally, a multicenter study would enhance the generalizability and external validity of the LCT predictive model by incorporating diverse patient populations and varying clinical practices.

## 5. Conclusion

In conclusion, we identified MRI-based imaging lesion features and elevated inflammatory markers in LCT. Based on these findings, we developed and validated a predictive model for LCT pain risk. We discovered that the lesion area can effectively predict the pain risk associated with LCT. These results enhance our understanding of LCT and can assist in individual clinical decision-making.

## Figures and Tables

**Table 1:** Demographic, imaging, and clinical features of LCT participants.

Variables	LCT (*n* = 35)	High-risk group (*n* = 20)	Low-risk group (*n* = 15)	*p* value
Age (years)	49.14 (16.06)	47.80 (14.56)	50.93 (17.71)	0.581
Sex				
Male	22 (62.86%)	14 (70.00%)	8 (53.33%)	0.313
Female	13 (37.14%)	6 (30.00%)	7 (46.67%)	
Height (cm)	169.63 (7.44)	170.15 (7.97)	168.93 (6.61)	0.644
Weight (kg)	68.00 (58.00, 74.00)	70.00 (61.00, 75.00)	64.00 (60.00, 70.00)	0.376
Imaging features				
Lesion width (mm)	6.13 (2.19)	7.27 (1.94)	4.61 (1.49)	< 0.001
Lesion length (mm)	64.00 (15.14)	72.72 (11.32)	52.38 (11.32)	< 0.001
Lesion circumference (mm)	134.52 (33.02)	151.84 (23.91)	111.44 (29.14)	< 0.001
Lesion area (mm^2^)	230.64 (115.14)	299.39 (94.23)	138.98 (66.27)	< 0.001
Clinical features				
White blood cell count (10^9^/L)	10.00 (2.36)	10.91 (2.19)	8.80 (2.01)	0.007
Neutrophils count (10^9^/L)	6.93 (5.51, 8.33)	8.03 (6.93, 8.68)	5.67 (5.34, 5.92)	0.005
Lymphocytes (10^9^/L)	1.77 (1.13, 2.21)	1.78 (1.39, 2.41)	1.49 (1.05, 1.89)	0.095
Eosinophils (10^9^/L)	0.02 (0.00, 0.03)	0.02 (0.00, 0.030)	0.02 (0.00, 0.03)	0.892
Basophils (10^9^/L)	0.02 (0.020, 0.03)	0.02 (0.020, 0.03)	0.02 (0.020, 0.03)	1.000
hsCRP (mg/L)	8.05 (4.33, 17.60)	13.54 (8.05, 29.59)	5.50 (3.72, 6.93)	0.002
IL-6 (pg/mL)	13.70 (9.82, 20.58)	17.45 (13.22, 24.10)	11.03 (7.96, 12.57)	0.003
Duration (days)	8.00 (6.00, 12.00)	12.00 (10.00, 13.00)	6.00 (5.00, 7.00)	< 0.001
Pain VAS score	4.00 (3.00, 6.00)	6.00 (5.00, 7.00)	3.00 (3.00, 3.00)	< 0.001
Typical symptoms				
Neck pain	35 (100)	20 (100)	15 (100)	—
Neck stiffness	24 (68.57)	15 (75.00)	9 (60.00)	0.344
Odynophagia	16 (45.71)	9 (60.00)	6 (40.00)	0.557

*Note:* Data are shown as *n* (%), median (IQR), and mean (SD).

Abbreviations: eGFR, estimated glomerular filtration rate; hsCRP, high-sensitivity C-reactive protein; IL-6, interleukin-6; LCT, longus colli tendinitis; VAS, visual analog scale.

**Table 2:** Univariate logistic regression analysis of risk factors for high-risk outcomes in LCT.

Variables	Univariate analysis		
	OR	95% CI	*p* value
Lesion width (mm)	2.72	(1.35, 5.48)	0.005
Lesion length (mm)	1.19	(1.05, 1.34)	0.005
Lesion circumference (mm)	1.06	(1.02, 1.10)	0.004
Lesion area (mm^2^)	1.03	(1.01, 1.05)	0.003
White blood cell (10^9^/L)	1.62	(1.09, 2.42)	0.017
Neutrophils (10^9^/L)	1.75	(1.12, 2.74)	0.014
hsCRP (mg/L)	1.14	(1.00, 1.31)	0.054
IL-6 (pg/mL)	1.05	(0.98, 1.13)	0.143

Abbreviations: CI, confidence interval; hsCRP, high-sensitivity C-reactive protein; IL-6, interleukin-6; LCT, longus colli tendinitis; OR, odds ratio.

**Table 3:** Multivariate logistic regression analysis of risk factors for high-risk outcomes in LCT.

Variables	*β*	SE	OR	95% CI	*p* value
Lesion area (mm^2^)	0.03	0.01	1.04	(1.01, 1.07)	0.014
Neutrophils (10^9^/L)	0.48	0.29	1.62	(0.99, 3.31)	0.100

Abbreviations: CI, confidence interval; LCT, longus colli tendinitis; OR, odds ratio; SE, standard error.

## Data Availability

The data that support the findings of this study are available from the corresponding authors upon reasonable request.

## Supporting Information

Supporting Table S1 provides detailed MRI acquisition parameters.

## References

[1] Lim W. Q., Ho E. C. (2022). Longus Colli Tendinitis: Acute Neck Pain With Retropharyngeal Swelling. *BMJ Case Reports*.

[2] Suh B., Eoh J., Shin J. (2018). Clinical and Imaging Features of Longus Colli Calcific Tendinitis: an Analysis of Ten Cases. *Clinical Orthopaedic Surgery*.

[3] Hartley J. (1964). Acute Cervical Pain Associated With Retropharyngeal Calcium Deposit: A Case Report. *The Journal of Bone and Joint Surgery*.

[4] Ring D., Vaccaro A. R., Scuderi G., Pathria M. N., Garfin S. R. (1994). Acute Calcific Retropharyngeal Tendinitis. Clinical Presentation and Pathological Characterization. *The Journal of Bone and Joint Surgery*.

[5] Igami E., Fukae J., Kanazawa K. (2022). Two Rare Diseases, Acute Calcific Retropharyngeal Tendinitis, and Crowned Dens Syndrome, Mimicking Meningitis: A Case Report. *Frontiers in Neurology*.

[6] Mohyeldin M., Singh H., Shrestha M., Leitao M., Kumar J. (2024). Acute Calcific Tendinitis of the Longus Colli: A Case Report and Review of the Literature. *Cureus*.

[7] Horowitz G., Ben-Ari O., Brenner A., Fliss D. M., Wasserzug O. (2013). Incidence of Retropharyngeal Calcific Tendinitis (Longus Colli Tendinitis) in the General Population. *Otolaryngology–Head and Neck Surgery*.

[8] Tagashira Y., Watanuki S. (2015). Acute Calcific Retropharyngeal Tendonitis. *Canadian Medical Association Journal*.

[9] Naqshabandi A. M., Srinivasan J. (2011). Teaching Neuroimages: Acute Calcific Tendinitis of Longus Colli Mimicking Meningismus. *Neurology*.

[10] Matsumoto Y., Nishioka H. (2024). Acute Calcific Tendinitis of the Longus Colli Muscle. *International Journal of Rheumatic Diseases*.

[11] Reitz I., Allen C., Rappaport D. E. (2023). An Unusual Cause of Fever, Neck Pain, and Neck Stiffness: Acute Calcific Tendinitis of the Longus Colli Muscle. *Journal of Emergency Medicine*.

[12] Filipovic T., Avsenik J. (2023). Retropharyngeal Calcific Tendinitis in the Neurological Emergency Unit, Report of Three Cases and Review of the Literature. *Radiology and Oncology*.

[13] Bannai T., Seki T., Shiio Y. (2019). A Pain in the Neck: Calcific Tendinitis of the Longus Colli Muscle. *The Lancet*.

[14] Gacouin A., Camus C., Le Tulzo Y. (2004). Assessment of Peri-Extubation Pain by Visual Analogue Scale in the Adult Intensive Care Unit: A Prospective Observational Study. *Intensive Care Medicine*.

[15] Park R., Halpert D. E., Baer A., Kunar D., Holt P. A. (2010). Retropharyngeal Calcific Tendinitis: Case Report and Review of the Literature. *Seminars in Arthritis and Rheumatism*.

[16] Gu W., Zhang M., Liang C. (2024). Nomogram to Estimate the Risk of Chronic Kidney Disease-Associated Pruritus in Patients With End-Stage Renal Disease Undergoing Peritoneal Dialysis: Model Development and Validation Study. *Blood Purification*.

[17] Langner S., Ginzkey C., Mlynski R., Weiss N. M. (2020). Differentiation of Retropharyngeal Calcific Tendinitis and Retropharyngeal Abscess: A Case Series and Review of the Literature. *European Archives of Oto-Rhino-Laryngology*.

[18] Roldan C. J., Carlson P. J. (2013). Longus Colli Tendonitis, Clinical Consequences of a Misdiagnosis. *The American Journal of Emergency Medicine*.

[19] Narváez J., Morales-Ivorra I., Martínez-Yelamos S., Narváez J. A. (2018). Acute Calcific Tendinitis of the Longus Colli Muscle. *The American Journal of Medicine*.

[20] Zapolsky N., Heller M., Felberbaum M., Rose J., Steinberg E. (2017). Calcific Tendonitis of the Longus Colli: An Uncommon but Benign Cause of Throat Pain That Closely Mimics Retropharyngeal Abscess. *Journal of Emergency Medicine*.

[21] Paik N. C., Lim C. S., Jang H. S. (2012). Tendinitis of Longus Colli: Computed Tomography, Magnetic Resonance Imaging, and Clinical Spectra of 9 Cases. *Journal of Computer Assisted Tomography*.

[22] Artenian D. J., Lipman J. K., Scidmore G. K., Brant-Zawadzki M. (1989). Acute Neck Pain due to Tendonitis of the Longus Colli: CT and MRI Findings. *Neuroradiology*.

[23] Bhatt A. A. (2018). Non-Traumatic Causes of Fluid in the Retropharyngeal Space. *Emergency Radiology*.

[24] Vollmann R., Hammer G., Simbrunner J. (2012). Pathways in the Diagnosis of Prevertebral Tendinitis. *European Journal of Radiology*.

[25] Hammer G. P., Vollmann R., Tomazic P. V., Simbrunner J., Friedrich G. (2012). Prevertebral Tendinitis: How to Avoid Unnecessary Surgical Interventions. *The Laryngoscope*.

[26] Matsuura H., Sugimoto Y., Sasaki E., Kiura Y., Kishida M. (2019). Acute Calcific Retropharyngeal Tendinitis. *Postgraduate Medical Journal*.

[27] Widlus D. M. (1985). Calcific Tendonitis of the Longus Colli Muscle: A Cause of Atraumatic Neck Pain. *Annals of Emergency Medicine*.

[28] Hunter C. A., Jones S. A. (2015). IL-6 as a Keystone Cytokine in Health and Disease. *Nature Immunology*.

[29] Tutar S., Ulusoy O., Ozturk E., Mutlu A., Saglam M. (2016). Calcific Tendinitis of the Longus Colli Muscle: A Rare Cause of Neck Pain. *Neurology India*.

[30] Zibis A. H., Giannis D., Malizos K. N., Kitsioulis P., Arvanitis D. L. (2013). Acute Calcific Tendinitis of the Longus Colli Muscle: Case Report and Review of the Literature. *European Spine Journal*.

