# Supplementary material for: Longus Colli Tendinitis: Analysis of MRI and Clinical Features With Predictive Pain Risk Model Development
Source: Pain Res Manag. 2025 Sep 11;2025:9211904. doi: 10.1155/prm/9211904 (PMC12446589; doi:10.1155/prm/9211904)
Supplement: Supporting Information — Additional supporting information can be found online in the Supporting Information section. [file 9211904.f1.docx]

**Table S1** MRI Acquisition Parameters

| **Sequence** | **Plane** | **TR (ms)** | **TE (ms)** | **Slice Thickness (mm)** | **FOV (mm)** | **Matrix** | **Voxel Size (mm)** | **Acquisition Time** |
| --- | --- | --- | --- | --- | --- | --- | --- | --- |
| T1WI TSE | Sagittal | 400 | 8.3 | 3 | 230×230 | 256×320 | 0.9×0.72×3 | 1 min 9 s |
| T2WI TSE | Sagittal | 3000 | 106 | 3 | 230×230 | 320×320 | 0.72×0.72×3 | 1 min 21 s |
| T2WI TSE | Transaxial | 4200 | 96 | 3 | 200×200 | 205×256 | 0.98×0.78×3 | 1 min 5 s |
| T2WI DIXON TSE | Sagittal | 3000 | 117 | 3 | 230×230 | 256×256 | 0.9×0.9×3 | 1 min 15 s |

Notes: T1WI, T1-weighted imaging; T2WI, T2-weighted imaging; TSE, turbo spin echo; TR, repetition; TE, echo time; FOV, field of view.
